# Coercion in psychiatric care: Global and Indian perspective

**DOI:** 10.4103/0019-5545.70971

**Published:** 2010

**Authors:** Ruchita Shah, Debasish Basu

**Affiliations:** Department of Psychiatry, Postgraduate Institute of Medical Education and Research (PGIMER), Chandigarh, India

## INTRODUCTION AND HISTORICAL ASPECTS

Coercion in health care is well known and forced treatment against the patient’s will is even more common in psychiatric care. In fact, psychiatry is the only discipline where coercion in care can be legal and state-sanctioned. Although autonomous decision-making is challenged because of the power relationship between the skilled practitioner and the unskilled patient; this power gradient is even more intense in psychiatry because of the nature of mental illness.[1] History is replete with examples of political abuse of the power entrusted in physicians and more specifically, psychiatrists, as seen during the Nazi rule and the Soviet regime when political dissidents were labeled ‘mentally ill’ and subjected to inhumane ‘treatments’. Coercion in psychiatric care is seen in the form of involuntary admission, involuntary treatment, seclusion/restraint, outpatient commitment, and in the Indian context, also includes surreptitious treatment. Thus to say the least, discussing the controversies hovering coercion in psychiatric care is of utmost importance and these controversies mainly center on the issues of ‘justification’ and ‘human rights’.

## CONCEPTUAL ISSUES

Coercion and persuasion are closely linked, but are definitely different terms. Persuasion is defined as the clinician’s aim ‘to utilize the patient’s reasoning ability to arrive at a desired result’,[2] whereas, coercion occurs ‘when the doctor aims to manipulate the patient by introducing extraneous elements which have the effect of undermining the patient’s ability to reason’.[3]

The four basic ethical principles[2] that dictate professional behavior are: (a) Respect for autonomy: this includes components of liberty or independence from controlling influences and agencies or the capacity for intentional action; (b) Beneficence: it refers to a moral obligation to act for the benefit of others; (c) Nonmaleficence: this simply means ‘First do no harm’; and (d) Justice: it refers to the fair and equitable distribution of treatment resources. Although these are the guiding principles, it is common in actual practice that these come in conflict with each other. The doctrine of double effect states that an action producing both helpful and harmful effects is not necessarily wrong. Although the deontological view states that right behavior (respect for autonomy) is obligatory without regard for consequences, paternalism is widely practiced in psychiatry. Paternalism is defined as the intentional overriding of one person’s known preferences or actions by another person, where the person who overrides justifies the action by the goal of benefiting or avoiding harm to the person whose preferences or actions are overridden.[2]

To promote individual autonomy and to encourage rational decision-making, the concept of informed consent[4] was developed. Informed consent is built upon the elements of information, decisional capacity, and voluntarism. Decisional capacity or competency, in turn, comprises of the ability to communicate, understand, and logically work with information and to appreciate the meaning of a decision within the context of one’s life. Competency assessment involves assessment of the mental ability to understand the nature and consequences of a decision (includes benefits/risks of consenting as well as refusing). Voluntarism[1] encompasses an individual’s ability to act in accordance with one’s authentic sense of what is good, right, and best in light of one’s situation, values, and prior history. Voluntarism further entails the capacity to make this choice freely and in the absence of coercion.

## PERCEPTION OF COERCION

It is widely believed by the providers that coercion is returned by patient’s subsequent gratitude for the psychiatrist’s unilateral action; the so called ‘Thank you’ test. A review by Katsakou *et al*.[5] found that between 33 and 81% of patients retrospectively regard their involuntary admission as justified or the treatment as beneficial. However, although some studies support this view others contest it. According to a study by Eriksson and Westrin,[6] about two-thirds of the committed versus nearly one-third of the voluntarily admitted patients reported the occurrence of coercive measures during the index period of care. Almost half of the patients (51% of the committed patients and 38% of the voluntarily admitted patients) stated at follow-up interviews that, during the index period of care, they had been violated as a person. Even voluntarily admitted patients often reported that they had been persuaded or pressurized by the caretakers or professionals to seek help.[7] Approximately a third of the patients who receives electroconvulsive therapy (ECT) perceive themselves to have been coerced into having the treatment. Even when patients are not given ECT under compulsion, they often feel that they did not freely give their consent.[8] The MacArthur Coercion Study[9] found that some patients’ views about the need for hospitalization, however, do change over time. Approximately half the patients who initially denied the need for hospitalization acknowledge such a need in retrospect. The amount of coercion that the individual experiences is strongly affected by the kind of pressures that others apply on him; the use of ‘negative’ pressures (threats and force) engender feelings of coercion; the use of ‘positive’ pressures (persuasion and inducements) do not. It is also related to a patient’s belief about the justice of the process by which he or she was admitted (procedural justice). That is, a patient’s beliefs that others acted out of genuine concern, treated the patient respectfully and in good faith, and afforded the patient a chance to tell his or her side of the story, are associated with low levels of experienced coercion. This is true for both voluntary and involuntary patients. Also, in a recent study,[10] when recruiting 115 patients who lacked the capacity to make treatment decisions, it was found that most people (83%) who regained the capacity following psychiatric treatment gave retrospective approval, even if the initial treatment wishes were overridden. These findings were similar in both informally and involuntarily admitted patients.

## CLINICAL, LEGAL, AND ETHICAL CONTROVERSIES

For legal sanction of involuntary admission, two elements are required: presence of a severe mental disorder that deprives the individual of the capacity to make treatment decisions and the likelihood of harm to self or others. The latter is termed the ‘dangerousness standard’. The Government has a duty toward every citizen, which includes ‘Police power’, that is, protecting each citizen from another persons’ injurious actions, and ‘Parens patriae power’, that is, the power and duty to protect individuals who cannot do so themselves. However, the biggest legal controversy is that the Governments also have a duty to protect the fundamental rights of any citizen, which includes liberty, and this would be jeopardized by involuntary admission. Also, individuals with mental illness are considered to have the capacity to consent to treatment including ECT, unless evidence to the contrary is compelling. The presence of psychosis, irrational thinking, or involuntary hospitalization does not in itself constitute proof of lack of capacity.

Ethically, involuntary admission may be justified, as the principle of beneficence directs physicians and others to care for individuals incapable of caring for themselves. Moreover, the proponents claim that involuntary hospitalization may restore autonomy to the mentally ill through treatment. However, this proposition lacks adequate evidence. Also, involuntary hospitalization is considered a clear infringement of a person’s autonomy and self-determination. In addition, the patient may have different belief systems for causation and treatment, and involuntary treatment hampers the right to refuse. The use of seclusion/restraint as a means of punishment or retribution for agitated, demanding or disruptive patients; or for convenience of staff (including standing or as-needed order) or as a substitute for treatment programs is absolutely unethical. The Erwady tragedy[11] in which the chained inmates died due to fire in a mental asylum in Tamil Nadu exemplifies the horrifying use of restraint.

Evidence has been gathered, which supports the clinical justification for involuntary admission and treatment under specific circumstances and otherwise unavoidable situations. Involuntary treatment was successful for patients with anorexia nervosa[12] and substance abuse.[13] In light of compromised autonomy that individuals in the throes of addiction exhibit, coercion may be necessary to initiate treatment, through an organized intervention or other direct confrontation.[13] A review of 18 studies on the outcome of involuntary hospital admissions demonstrated that most involuntarily admitted patients showed substantial clinical improvement over time.[5] Acute involuntary hospitalization may not be automatically associated with a higher risk for overall negative outcome.[14] Seclusion/restraint may be beneficial as it prevents imminent harm to the patient or other persons when other means of control are not effective or appropriate. Also, association between perceived coercion at the time of hospital admission and patient adherence to medication and other treatment after discharge is not always found. Although some have proposed that coercive treatment can lead to the development of post traumatic stress disorder among patients with schizophrenia, many have found that schizophrenic and delusional symptoms are more traumatic than the coercive measures used to control them. Nonetheless, most have warned about the risk of physical harm if patients are not monitored adequately when under seclusion/restraint orders. Also, there has been a shift in the dangerousness criteria from ‘hard’ to ‘soft’. ‘Arousing aggression’, ‘severe self-neglect,’ and ‘severe social breakdown’ are applied to psychotic patients and these are cited as reasons for emergency involuntary admissions.

In terms of involuntary pharmacological treatment, depots are perceived as more coercive than oral anti-psychotics and are associated with a relative lack of true autonomy.[15] However, use of depot preparation is associated with enhanced compliance.[16]

In case of ECT, some strongly contend that true informed consent is ‘nonexistent,’ as the ‘harmful effects’ of this treatment are minimized in the informed consent process. According to Andrade,[17] the risk-benefit ratio should be weighed for involuntary use of ECT (with consent from surrogate decision-maker) based on efficacy, side effects, and feasibility of other alternatives.

## INDIAN PERSPECTIVE REGARDING COERCION IN PSYCHIATRIC CARE

### Ethical issues

Few authors have tried to examine ethical issues in relation to psychiatric care in India.[18] Individual autonomy is valued in Scandinavian, European, and American cultures, but is not empowering for the traditional, family-centered societies in Indian, Arab, African, and Japanese cultures. This difference may affect the use of involuntary admission and informed consent, among other practices, in traditional versus Western societies.[3,19] In addition, medical paternalism (‘the doctor knows the best’) is more acceptable to the patient in these societies. However, that does not automatically and necessarily imply the right of the treating agency to assume the role of a ‘do-gooder’ in the form of involuntary treatment and coercion. Furthermore, with the rapidly changing socioeconomic, cultural, and psychosocial profiles of the traditional rural-oriented and family-centered societies of India and the Asian countries in general, it is all the more important to be aware of the individual rights and preferences regarding the necessity, mode, and venue of psychiatric treatment, along with those of the family members.

### Clinical issues

A recent study in Vellore, India[20] found that more than half of the patients were not aware of the details of ECT, even at the end of the course, but were not unhappy about receiving ECT. The relatives knew of its benefits and risks and felt that they were offered a choice of treatment, but also admitted to feeling coerced.

In a study in an urban Indian population, families in half the cases of patient noncompliance administered medication to them without the patients’ knowledge (surreptitious treatment), under the supervision of the psychiatrist.[21] Thus, individual autonomy and independence may not equally generalize to cultures where familial interdependence is stronger and collective goals of the family are dominant. According to Srinivasan and Thara,[21] principles of beneficence and utilitarianism are addressed here, as the treatment helped many patients recover from the illness, enough to voluntarily participate in further treatment, without many negative effects and at a low cost. Thus, they benefited both financially and emotionally. However, the deception of concealed medicines involves manipulation of persons – albeit for benevolent ends – which denies their dignity, by depriving them of the right to know what is being done to their minds and bodies, and also, their right to refuse is violated.

Although concealed medicines possibly have the advantage of avoiding physical harm if a patient is expected to violently resist taking medicine, the dangers are ample, including the possibility of serious side effects. Without informing their family members, patients may consume other medications or psychoactive substances that interfere with the therapeutic effects of the concealed medication or that are otherwise problematic. Periodic monitoring would be difficult as patient co-operation for investigations cannot be enlisted. Also, the actual motive of the family may be harmful to the patient.

### Legal issues

Refusal to consent and incompetency to consent are both included in the section on ‘admission under special circumstances’ in the Mental Health Act (MHA) 1987, of India.[22] It has been strongly criticized that ‘competence’ has not been defined in the purview of the MHA.[23] In MHA, there is no separate provision for forced treatment. Involuntary treatment thus is presumed to be subsumed under admission under special circumstances and involuntary admission. As a ‘mentally ill’ person is defined as a person who needs treatment because of his mental disorder, then it can be extrapolated that as per Sections 19 and 20, it becomes the psychiatrist’s duty to treat the person. In other words, the clauses of the Act do not make the situation any better or simpler. Specific guidelines and criteria as to when and under what circumstances involuntary treatment or admission is justified are woefully missing from the existing MHA.

The draft proposing amendments[24] in the MHA 1987, classifies admissions as being ‘independent’ where the person can decide for himself or herself, without support or requires minimal support, and ‘supported’ admissions are where the persons needs substantial or high support, approaching 100% support; although it remains vague about provisions for assessing and implementing the same. Also, it mentions that high levels of support bordering on 100% support are to be viewed as a temporary phenomena and as soon as the person is judged to be able to make independent decisions, he or she to be allowed to decide for himself or herself. This proposed provision may help to strike a balance between beneficence and right to autonomy. The Indian Psychiatric Society is currently in the process of finalizing its comments on the draft MHA amendment document. Although premature to affirm at this moment, it is expected and desirable that this consultative process would contribute to further improvement in the situation as far as coercive practices in psychiatric care are concerned.

## TENTATIVE ANSWER TO THE DILEMMAS

Although there are no easy answers (one would be surprised if there were any!), one of the most crucial elements of current psychiatric care lies in the thorough assessment of the capacity of decision-making and not a mere assumption of lack of capacity just because the patient has a mental illness. The decision-making capacity can be improved by improving the information through repeated disclosure of information, group sessions, videotapes and computer programs, and involvement of family members. To the extent possible, the patient (and family) should be involved or at least made aware of the treatment and the targets to be achieved by it. The major instrument for achieving this is communication: skilled communication that is two-way, open, repeated, empathic, and accommodative. Along with communication, detailed documentation is necessary to explain why a particular action (e.g., involuntary treatment or seclusion/restraint, etc.) was felt necessary under the specific circumstances. Advanced planning for the possibility of future incapacity, by use of joint crisis plans reduces compulsory admissions and treatment in patients with severe mental illness, and may affect the amount of perceived coercion. The doctrine of ‘least restrictive alternative’ (‘least’ in terms of modality, severity, and duration of the action taken) should be used. Positive approaches, such as persuasion, should be the strategies of choice and negative approaches, such as threats should be avoided. One should be explicit about what one is doing and why, allow patients to tell their side of the story, and seriously consider this information. The last, and possibly one of the most relevant dictums is discussion among colleagues for the most acceptable approach. Finally, it is obvious that more research is needed in this area, particularly from India and the other Asian and developing countries. Such research needs to be not only service- and doctor-oriented, but also patient- and society-oriented, so as to enable each side to hear the voice of the other.
